# Mesenchymal stem cell secretome-loaded fibrin glue improves the healing of intestinal anastomosis

**DOI:** 10.3389/fbioe.2023.1103709

**Published:** 2023-03-31

**Authors:** Wenwen Yu, Haicun Zhou, Xueliang Feng, Xiaoqin Liang, Dengwen Wei, Tianhong Xia, Bin Yang, Long Yan, Xiaochen Zhao, Hongbin Liu

**Affiliations:** ^1^ The Second Clinical Medical College, Lanzhou University, Lanzhou, China; ^2^ Department of Breast Surgery, Gansu Maternal and Child Healthcare Hospital, Lanzhou, China; ^3^ Department of Abdominal Surgery, Gansu Provincial Cancer Hospital, Gansu Provincial Academic Institute for Medical Research, Lanzhou, China; ^4^ Key Laboratory of Stem Cells and Gene Drugs of Gansu Province, The 940th Hospital of Joint Logistics Support Force of Chinese People’s Liberation Army, Lanzhou, China

**Keywords:** stem cell secretome, fibrin glue, anastomotic healing, cell-free therapy, drug delivery system, regenerative medicine

## Abstract

Anastomotic leakage is a serious complication following gastrointestinal surgery and one of the leading causes of patient mortality. Despite the significant clinical and economic burden, there are currently no reliable treatment options to improve the healing of intestinal anastomosis and subsequently prevent anastomotic leakage. Recently, the development of regenerative medicine has shown promise for improving anastomotic healing. Recent studies have illustrated that stem cell-derived secretome can enhance tissue regeneration without the safety and ethical limitations of stem cell transplantation. Herein, we developed a fibrin glue topical delivery system loaded with mesenchymal stem cells (MSCs)-derived secretome for controlled delivery of bioactive factors, and evaluated its application potential in improving the healing of intestinal anastomosis. Under *in vitro* conditions, the MSCs secretome significantly promoted cell proliferation viability in a dose-dependent manner and resulted in the controlled release of growth factors *via* fibrin glue delivery. We established a rat surgical anastomotic model and experimentally found that MSCs secretome-loaded fibrin glue enhanced anastomotic bursting pressure, increased granulation tissue formation and collagen deposition, and significantly promoted anastomotic healing. Mechanistically, fibrin glue accelerated cell proliferation, angiogenesis, and macrophage M2 polarization at the surgical anastomotic site by releasing bioactive factors in the secretome, and it also alleviated the inflammatory response and cell apoptosis at the anastomotic site. Our results demonstrated for the first time that MSCs-derived secretome could promote the healing of intestinal anastomosis. Considering the accessibility and safety of the cell-free secretome, we believed that secretome-loaded fibrin glue would be a cell-free therapy to accelerate the healing of intestinal anastomosis with great potential for clinical translation.

## 1 Introduction

Anastomotic leakage is a serious clinical syndrome induced by the infiltration of intestinal contents into the abdominal cavity due to failed anastomotic healing following intestinal resection and anastomosis. It can easily progress to severe abdominal infection and sepsis, and it is also one of the main causes of death (Huang et al., 2020). It has been reported that the incidence of anastomotic leakage following gastroesophageal surgery is 7%–12%, and the incidence of that after colorectal surgery is 3%–19% (Khan et al., 2012; Wang et al., 2019). It is estimated that at least one million patients worldwide suffer from anastomotic leakage-related complications after surgery annually (Rose et al., 2015). With improved surgical techniques for gastrointestinal surgery, improved surgical instruments, and accumulated experience in perioperative management in recent years, the incidence and mortality of anastomotic leakage have been reduced (Reischl et al., 2021). However, studies have shown that still more than half of patients cannot benefit from these interventions (Khan et al., 2012; Kiran et al., 2015). Therefore, unfavorable healing of surgical anastomosis has been an intractable problem in clinical practice.

Stem cell-based regenerative therapies are considered a promising scheme as stem cells have the ability to differentiate into intestinal cells or promote intestinal cell proliferation (Flatres et al., 2019; Lightner, 2019; Kent et al., 2021; Zhang et al., 2022). Several preclinical studies investigated the role of stem cell transplantation in accelerating the healing of intestinal anastomosis, and preliminary results indicated that stem cell transplantation has the potential to protect surgical anastomosis (Maruya et al., 2017; Van de Putte et al., 2017; Sukho et al., 2018; Morgan et al., 2020; Pan et al., 2020). However, the potential tumorigenicity, immune intolerance, and other safety risks related to stem cell transplantation, coupled with the strict supervision of stem cell clinical trials by regulatory authorities, have hampered the clinical translation of stem cell therapies for tissue regeneration and repair (Deng et al., 2018; Nathan and Ustun, 2019). In recent years, de-cellular modalities associated with stem cells have attracted extensive attention. Mesenchymal stem cells (MSCs) can secrete numerous bioactive components such as cytokines, growth factors, exosomes, and even extracellular matrix (Lee et al., 2017; Rahimi et al., 2021; Chouw et al., 2022; Lei et al., 2022; Tang et al., 2022), collectively referred to as the secretome, and these bioactive factors and paracrine signaling molecules exert critical roles in tissue repair and regeneration. The secretome, which can be dissolved in a medium after secretion by MSCs *in vitro*, is thus also referred to as a “conditioned medium”. Multiple fundamental studies support the application of MSC-derived secretome in the reconstruction of injured organs or tissues (Rhatomy et al., 2020; Lin et al., 2021). Furthermore, clinical trials have shown that MSCs-derived secretome is promising in the treatment of hereditary pulmonary arterial hypertension (Hansmann et al., 2022), protection against skin aging (Sushmitha et al., 2018; Liang et al., 2022), and hair regeneration (Shin et al., 2018). Although the MSCs-derived secretome holds strong potential to promote regeneration and repair, unfortunately, there are currently no reports on its role in anastomotic healing.

**FIGURE 1:**
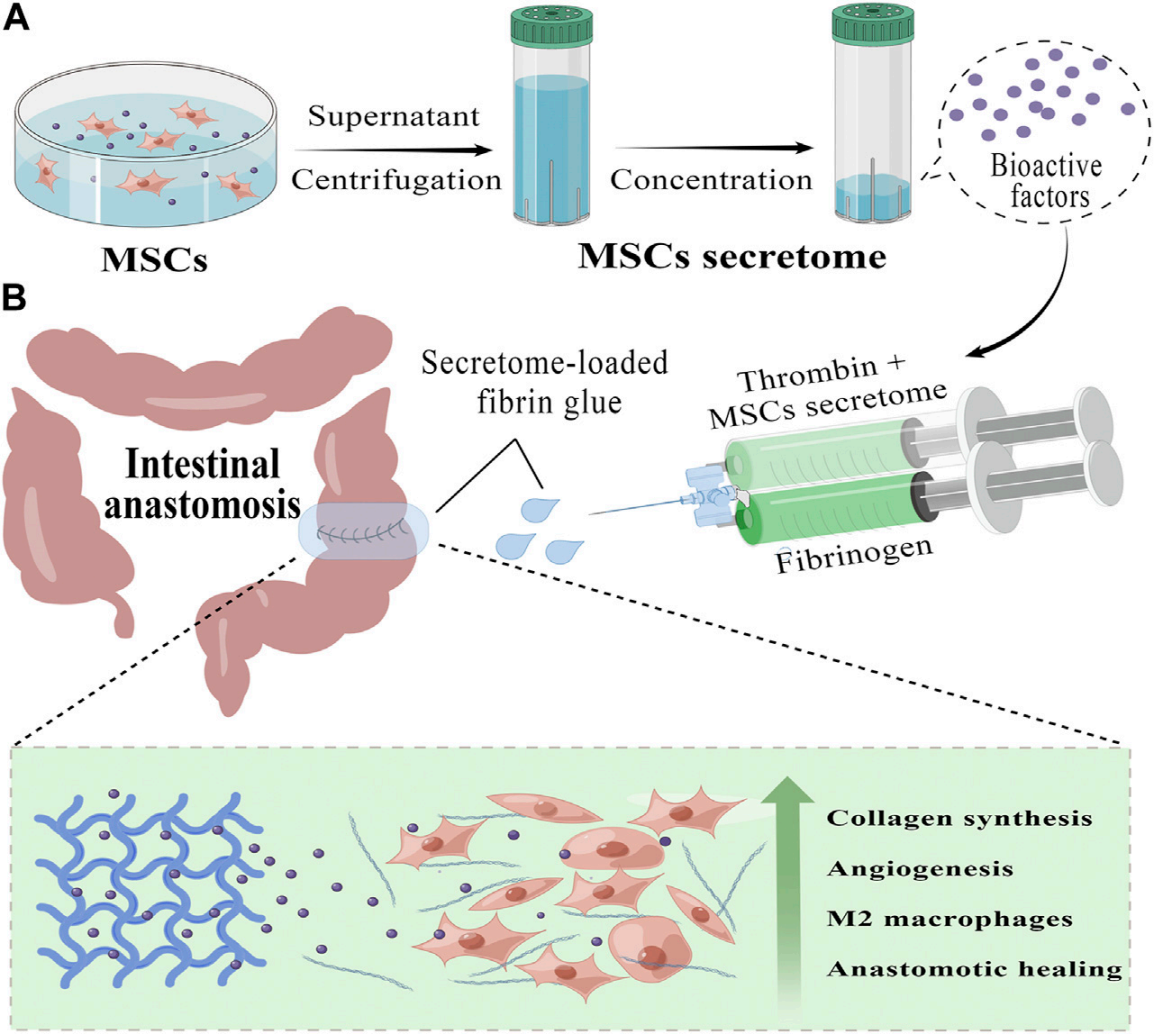
Preparation and application of MSCs secretome-loaded fibrin glue (by Figdraw). **(A)** We collect the secretome from the conditioned medium of human umbilical cord mesenchymal stem cells (hucMSCs) and further concentrate it into different secretome concentrates with protein ultrafiltration tubs, which contain a variety of bioactive factors that promote tissue repair. **(B)** Using a convenient mixed injection, we can rapidly form an MSCs secretome-loaded fibrin glue (Secretome/FG) patch covering the surgical anastomosis tightly. By slowly releasing bioactive factors, Secretome/FG promotes collagen synthesis and angiogenesis, regulates macrophage M2 polarization, and ultimately improves the healing of ischemic intestinal anastomosis.

In light of these findings, we aimed to assess the effect of MSCs secretome on anastomotic healing in a rat model. To ensure the local release of MSCs secretome at the anastomotic site, we incorporated MSCs secretome into an FDA-approved fibrin glue (FG) and developed a topical delivery system (Secretome/FG), which could rapidly form an intestinal patch through gelatinization at the anastomotic site (Figure 1). *In vitro* experiments revealed that the MSCs secretome could boost the viability of key cells involved in wound healing dose-dependently. In a rat model of anastomosis, MSCs secretome-loaded fibrin glue (Secretome/FG) significantly improved the healing of surgical anastomosis, which might provide a novel strategy to accelerate anastomotic healing, contributing to the clinical translation of MSCs secretome-based therapies for anastomotic repair.

## 2 Materials and methods

### 2.1 Cell culture

Human umbilical vein endothelial cells (HUVEC, passage 5) and endothelial cell medium were procured from Beijing BNCC Science & Technology Co., Ltd. (Beijing, China). Human intestinal epithelial cells (Caco-2), mouse macrophages (Raw264.7), and mouse fibroblasts (L929) were commercially available from Shanghai Fuheng Science & Technology Co., Ltd. (Shanghai, China) and cultured in high-glucose Dulbecco’s modified Eagle’s medium (DMEM) supplemented with 10% FBS (Gibco, Amarillo, United States). Human umbilical cord mesenchymal stem cells (hucMSCs), donated by the Key Laboratory of Stem Cell and Gene Drug of Gansu Province, were isolated from human umbilical cord tissues and cultured in low-glucose DME containing 10% FBS (Gibco). Cells were placed in standard cell culture flasks and cultured at 37°C with 5% CO_2_ and a relative humidity of 95%. The medium was renewed every 2-3 days, and upon reaching 80%-90% confluence, the cells were passaged using trypsin-EDTA (Solarbio, Beijing, China).

### 2.2 Preparation of MSCs secretome and Secretome/FG delivery system

Secretome was obtained from the conditioned medium of hucMSCs at passage 3–5. To obtain MSCs secretome, MSCs were plated at 2 × 10^6^ cells per 15 cm culture dish and cultured in DMEM containing 10% FBS until 70% confluence (approximately 0.9 × 10^6^ cells). 20 mL of serum-free medium (Gibco) without FBS was added to the MSCs and cultured for another 2 days (approximately 1.4 × 10^7^ cells and at least 90% live cells). The supernatant was collected and centrifuged (2000×g) to remove debris and dead cells. After the removal of impurities using a 0.22 μM syringe (Beyotime, Shanghai, China), the hucMSCs conditioned medium (secretome) was collected. Secretome was concentrated using Amnicon Ultra Centrifugal Filter Units with a molecular cutoff of 3 kDa (Millipore, Germany), and the concentrated secretome was preserved in a −80°C freezer for later use. For animal study, we used 40 μL of 25-fold concentrated secretome to ultimately obtain 100 μL of 10-fold concentrated secretome (approximately 1.2 mg/mL) in the mixture with fibrin glue for injection. Fibrin glue (Specification: 5 mL; Bioseal Biotech, Guangzhou, China) is mainly consisted of fibrinogen powder and thrombin-containing catalyst fines. To prepare Secretome/FG, the thrombin-containing catalyst fines were first incorporated into MSCs secretome solution to produce mixed MSCs secretome-catalyst suspension. Then, the fibrinogen powder was immersed in ultrapure water to generate a fibrinogen solution. Finally, MSCs secretome-catalyst suspension and fibrinogen solution were separately filled into the two chambers of a dual-chambered syringe (Bioseal Biotech) at a 1:1 ratio, which was then equipped with a droplet head (Bioseal Biotech). Using this syringe, the solution was directly pushed and smeared onto the surgical anastomosis.

### 2.3 Cell proliferation assay

To analyze the pro-proliferative ability of MSCs secretome *in vitro*, cell viability was quantified using a cell counting kit-8 (CCK-8; Yeasen Biotech, China) according to the manufacturer’s instructions. Briefly, MSCs were seeded into 96-well plates at a density of 1,500 cells per well and treated with 0-, 1.25-, 2.5-, 5-, 10-, and 20-fold concentrated MSCs secretome for 1, 2, and 3 days. At the end of the treatment, the medium was removed and 100 μL of medium containing 10% CCK-8 were added into each well and incubated for an hour at 37°C. The absorbance at 450 nm was measured using a microplate reader. Cell viability was calculated according to the formula {cell viability = [(As-Ab)/(Ac-Ab)]*100%}.

### 2.4 Measurement of growth factors

ELISA kits were employed to quantify cytokine concentrations according to the manufacturer’s protocol. Growth factors to be measured include human basic fibroblast growth factor (FGF-2; Jinglai Biotechnology, Shanghai, China), human vascular endothelial growth factor (VEGF; Jinglai Biotechnology), human insulin-like growth factor 1 (IGF-1; Jinglai Biotechnology), rat transforming growth factor-β1 (TGF-β1; Jinglai Biotechnology), rat VEGF (Jinglai Biotechnology), rat FGF-2 (Jinglai Biotechnology), rat interleukin-1β (IL-1β; Jinglai Biotechnology), rat tumor necrosis factor-α (TNF-α; Jinglai Biotechnology), rat interleukin 4 (IL-4; Jinglai Biotechnology), and rat interleukin 10 (IL-10; Jinglai Biotechnology).

### 2.5 Controlled release ability of MSCs secretome

To assess the release of growth factors (FGF-2, VEGF, and IGF-1) from Secretome/FG, their levels were quantified by ELISA. A 100 μL fibrin glue containing 10-fold MSCs secretome was incubated at 500 μL PBS (37°C, 5% CO_2_). On days 0.5, 1, 2, 3, 5, 7, 10, and 14, half of the PBS was collected. An equal volume of fresh PBS was added to the test tube for further detection. The concentration of FGF-2 released in the culture medium was measured using a human FGF-2 ELISA kit (Jinglai Biotechnology). The concentration of VEGF released in the culture medium was measured using a human VEGF ELISA kit (Jinglai Biotechnology). The concentration of IGF-1 released in the culture medium was measured with a human IGF-1 ELISA kit (Jinglai Biotechnology).

### 2.6 Animal experiments

Male Sprague-Dawley rats (200–220 g) were provided by the 940th Hospital of Joint Logistics Support Force of PLA and housed under a natural light/dark cycle at 25°C with free access to food and water. Rats were fasted from 12 h before surgery until 24 h after surgery. The rats were anesthetized with an intraperitoneal injection of 2% pentobarbital sodium (40 mg/kg), and their abdomens were shaved after anesthesia. Next, a laparotomy was performed using a sterile surgical technique. Through a careful exploration of the abdominal viscera, the ileum was dissected at 2 cm proximal to the cecum (ligation of the mesenteric vascular arch at the proximal and distal 0.5 cm of the ileal anastomosis before ileum dissection is essential to reduce bleeding and improve survival). After that, the broken ends of the intestinal tubes were sutured using a 6–0 absorbable surgical suture (Jinhuan, Shanghai, China), and then the ileum was ligated (anastomosis). We used 12 sutures for a full-thickness interrupted varus suture to construct the anastomosis to make it completely “sealed”. After anastomosis, to ensure no anastomotic leakage, we clipped the proximal and distal ends of the anastomosis simultaneously and injected normal saline into the vicinity of the anastomosis through a syringe fitted with a 26G needle (Shinva, Zhibo, China). No fluid outflow represented a successful surgical anastomosis. To testify to the therapeutic effect of MSCs secretome-loaded fibrin glue, rats were assigned into four groups according to different interventions, including the negative control group (NC; without any treatment after intestinal anastomosis), the MSCs secretome intravenous injection group (Secretome-iv; tail vein injection of 100 μL 10-fold MSCs secretome), the fibrin glue group (FG; smeared with 100 μL fibrin glue at surgical anastomosis), and the MSCs secretome-loaded fibrin glue group (Secretome/FG; smeared with 100 μL fibrin glue containing 10-fold MSCs secretome at surgical anastomosis). The abdomen was closed, and the rat was placed on a warm pad. The whole surgical process was done by three surgeons. The baseline conditions of rats were constantly observed after surgery, and the changes in the weight of feces and body weight were recorded. The rats were reanesthetized with sodium pentobarbital on day 7 following the operation. At first, the baseline conditions, such as intestinal adhesions, anastomotic abscesses, or infections in the abdominal cavity, were evaluated, and then the ileal anastomotic tissues were collected for further analysis. Lastly, the rats were euthanized with carbon dioxide.

### 2.7 Anastomotic bursting pressure assessment

The anastomotic bursting pressure is an important index reflecting the strength of anastomotic healing. After PBS washes, a 3 mm catheter was placed on one side of the isolated intestinal stump, and both sides of the stump were sutured with 3-0 silk sutures (Jinhuan) to close the lumen. The catheter was connected to an infusion syringe and a manometer, the isolated intestinal tract containing anastomosis was immersed in water, and air was injected into the intestinal tract using a syringe. The anastomotic bursting pressure was defined as the intraluminal pressure where air leakage was initially observed.

### 2.8 Histological analyses

On day 7 after surgery, the intestinal anastomotic tissues were collected and fixed with 4% paraformaldehyde (Servicebio, China) after PBS rinses. Next, the anastomotic tissues were embedded with paraffin and sliced into 5-μM-thick sections (surgical anastomotic tissues after repair) for later use. Histological sections were subjected to hematoxylin and eosin (HE) staining and Masson-Trichrome-Goldner (MTG) staining as per standard protocols to assess the level of inflammation, granulation tissue formation, and collagen expression in the anastomotic tissues.

Cell proliferation, angiopoiesis, and macrophage polarization in tissues were assessed by immunofluorescence. Sections were probed using Anti-CD86 (1:200; Affinity, Jiangsu, China), anti-CD206 (1:200; Affinity), anti-CD31 (1:500; Servicebio, Wuhan, China), anti-α-SMA (1:1,000; Servicebio), and anti-Ki-67 (1:1,000; Servicebio), followed by incubation with corresponding secondary antibodies at room temperature. Ultimately, the tissues were counterstained with DAPI. Images were recorded with a fluorescence microscope (OLYMPUS, 1X71 + DP71).

### 2.9 TUNEL staining

To assess the apoptosis in tissues surrounding the anastomosis, TUNEL staining was performed using a Fluorescein (FITC) Tunel Cell Apoptosis Detection Kit (Servicebio) according to the manufacturer’s instructions. The percentage of apoptotic cells was determined depending on the proportion of FITC- and DAPI-positive nuclei.

### 2.10 Statistical analysis

All experimental data were expressed as mean ± standard deviation and analyzed statistically by GraphPad Prism 8 and SPSS 25.0 software. Statistical group comparisons were done by one-way analysis of variance (ANOVA) and *t*-test accordingly. For all comparisons, *p*-values <0.05 were considered statistically significant.

## 3 Results

### 3.1 Identification of MSCs secretome

We characterized the key components and bioactivity of the extracted MSCs secretome. At first, we determined the expression of FGF-2, VEGF, and IGF-1, which have been previously reported to be expressed in the MSCs secretome (Chen et al., 2021; Liang et al., 2022). ELISA results showed that these factors were all expressed in the MSCs secretome. The concentrations of FGF-2, VEGF, and IGF-1 were 302.6 pg/mL, 580.01 pg/mL, and 714.05 pg/mL, respectively. Surprisingly, the aforementioned growth factors have also been reported to promote anastomotic repair (Oines et al., 2014; Li et al., 2017).

**FIGURE 2:**
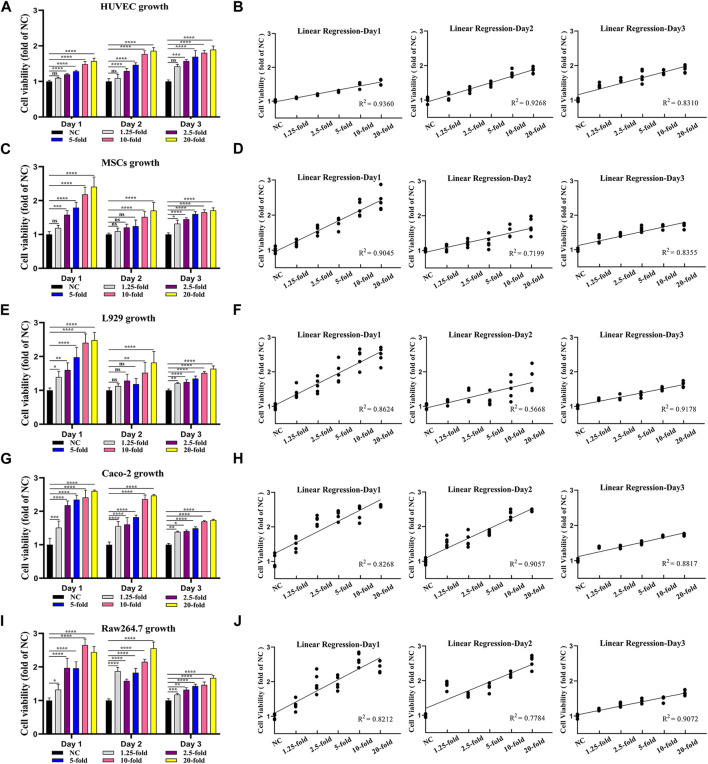
MSCs secretome enhances the viability of key cells for tissue healing in a dose-dependent manner. Vascular endothelial cells (HUVEC) **(A)**, mesenchymal stem cells (MSCs) **(C)**, fibroblasts (L929) **(E)**, intestinal epithelial cells (Caco-2) **(G)**, and macrophages (Raw264.7) **(I)** are treated with various MSCs secretome concentrations (1.25-, 2.5-, 5-, 10-, and 20-fold), and cell viability is measured using the CCK-8 assay on day 1, day 2, and day 3. The linear regression relation between MSCs secretome concentration and cell viability, including HUVEC **(B)**, MSCs **(D)**, L929 **(F)**, Caco-2 **(H)**, and Raw264.7 **(J)** (*n* = 5 per group; ANOVA). ****p < 0.0001, ***p < 0.001, **p < 0.01, *p < 0.05, ns ≥ 0.05.

Next, to determine the bioactivity of the extracted MSCs secretome, we tested the effect of MSCs secretome on cell viability by CCK-8 assay (Figures 2A, J). Five kinds of wound healing-related cell lines (vascular endothelial cells HUVEC, mesenchymal stem cells MSCs, fibroblasts L929, intestinal epithelial cells Caco-2, and macrophages Raw264.7) were cultured in the medium containing MSCs secretome at different concentrations (1.25-, 2.5-, 5-, 10-, and 20-fold), and the cell proliferation was assessed on days 0, 1, 2, and 3. Almost all cell lines treated with MSCs secretome showed significant increases in cell numbers on days 1, 2, and 3 compared with the non-treated cells (absence of MSCs secretome). Similarly, when comparing most groups on the same day, the higher the concentration of MSCs’ secretome, the greater the viability of cells (Figures 2A, C, E, G, I). To further investigate the relationship between MSCs secretome concentration and cell viability, we performed linear regression analysis. Linear regression results revealed a strong positive correlation between MSCs secretome concentration and cell viability, regardless of cell type (Figures 2B, D, F, H, J). These results indicated that the MSCs secretome could enhance cell viability in a dose-dependent manner.

Taken together, the above-mentioned results demonstrated that our collected MSCs secretome contained key growth factors and that the MSCs secretome could promote the viability of key cells involved in the healing process, showing good biological activity.

### 3.2 Secretome/FG could release the growth factors for up to 10 days

**FIGURE 3:**
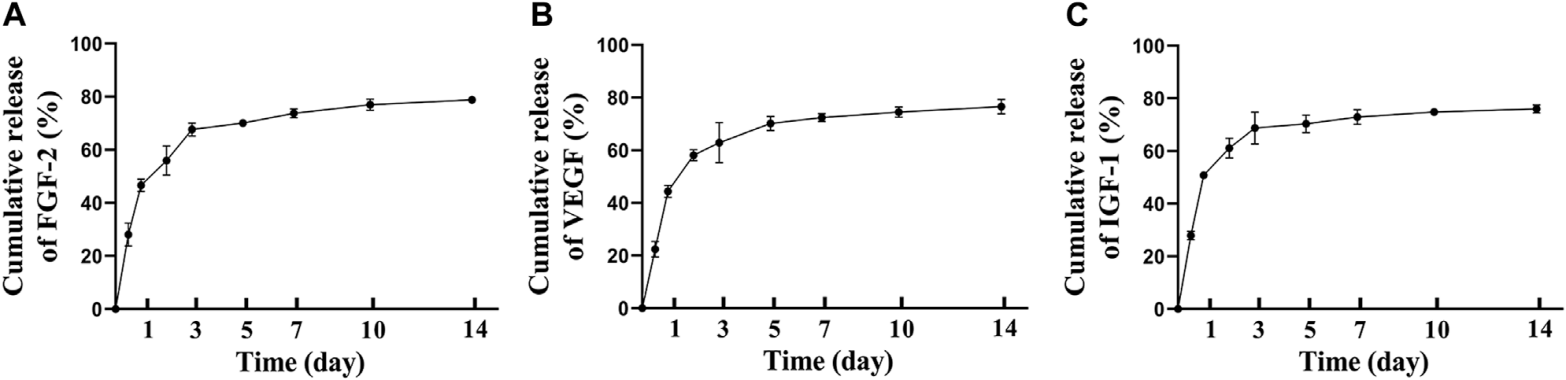
Controlled release of MSCs secretome from fibrin glue *in vitro*. The cumulative release of FGF-2**(A)**, VEGF**(B)**, IGF-1**(C)** from MSCs secretome-loaded fibrin glue; *n* = 3 per group.

After incorporating MSCs secretome into fibrin glue, we analyzed the release kinetics of MSCs secretome from Secretome/FG, and the release profiles of therapeutic proteins were measured using FGF-2, VEGF, and IGF-1 as model proteins. Based on ELISA results, the cumulative protein release curves of FGF-2 (Figure 3A), VEGF (Figure 3B), and IGF-1 (Figure 3C) all showed a rapid release in the first 3 days, followed by a sustained release period of up to 7 days. It was demonstrated that the Secretome/FG delivery system could control the sustained release of bioactive factors from the MSCs secretome.

### 3.3 Secretome/FG improves the anastomotic healing

**FIGURE 4:**
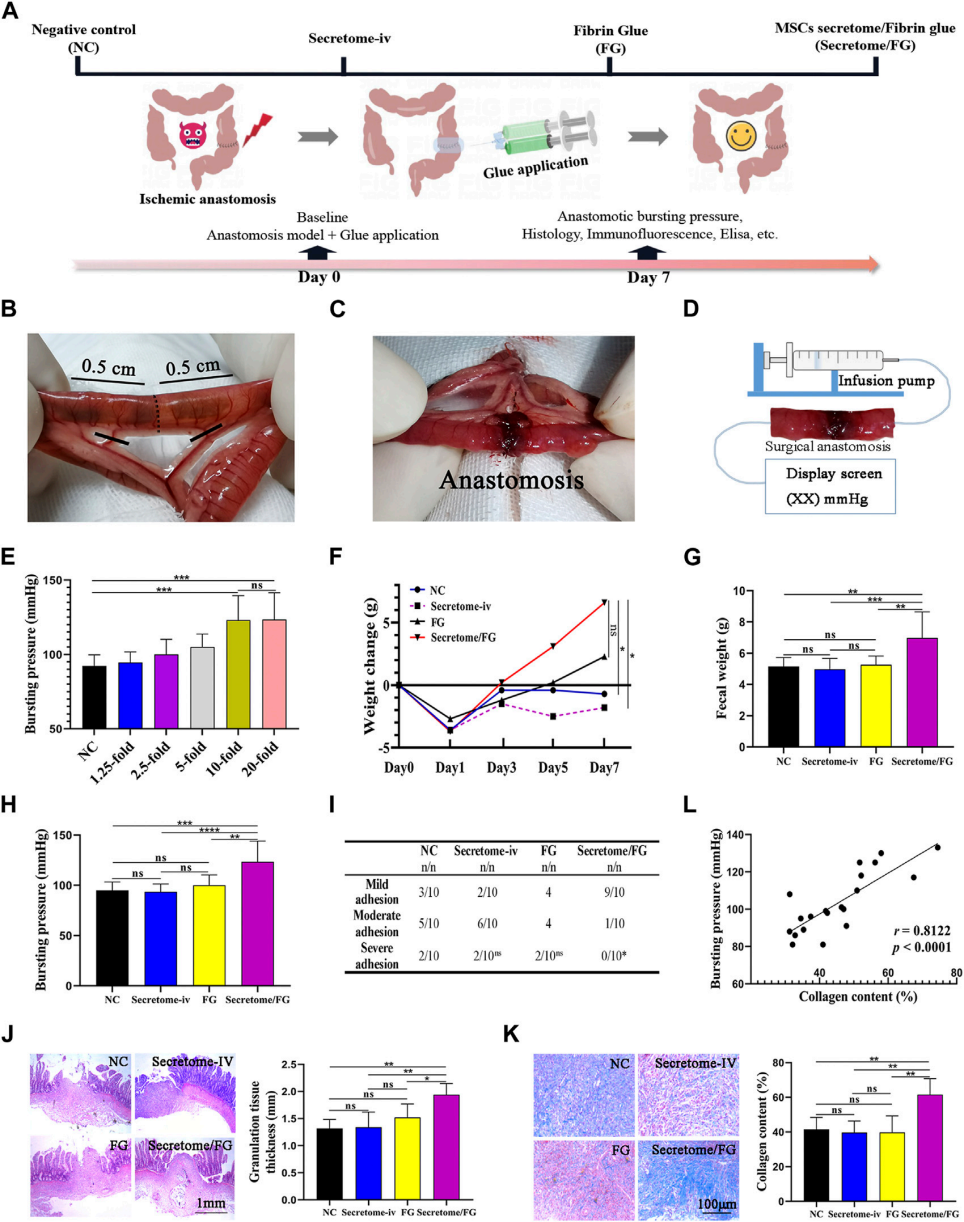
MSCs secretome-loaded fibrin glue (Secretome/FG) improves intestinal anastomosis healing in a rat model. **(A)** Schematic of groups and the experimental design for a 7 days animal study. **(B, C)** Schematic drawings of blood vessel ligation to induce ischemia in intestinal anastomosis. **(D)** The method for detecting anastomotic bursting pressure, which is the maximum pressure measured by a pressure transducer during the continuous infusion of air into the sealed isolated intestine containing surgical anastomosis. **(E)** Screening of optimal secretome concentration by bursting pressure analysis of intestinal anastomosis. Overall, MSCs secretome at a concentration of 10-fold is the optimal concentration to improve intestinal anastomosis healing (*n* = 10 per group; ANOVA). **(F, G)** The change in body weight and fecal weight indicates that the rats treated with Secretome/FG promoted a significant recovery of gastrointestinal function at POD 7 compared with the negative control group (NC) (*n* = 10 per group; ANOVA). **(H)** The comparison of anastomotic bursting pressure by different interventions (*n* = 10 per group; ANOVA). **(I)** The scoring analysis reveals that Secretome/FG reduced the abdominal adhesion on the 7th day after surgery compared with other interventions (*n* = 10 per group; Chi-Square Test). **(J, K)** The histological and quantitative analysis by HE and Masson staining shows that the repair of intestinal anastomosis depends on the regenerated granulation tissues for all interventions, in which the granulation tissues are the thickest with the most collagen content for the Secretome/FG treatment (*n* = 5 per group; ANOVA). **(L)** The correlation analysis of individual bursting pressure values with collagen content (*n* = 5 per group; Pearson’s correlation analysis). ****p < 0.0001, ***p < 0.001, **p < 0.01, *p < 0.05, ns ≥ 0.05.

We then hypothesized that MSCs secretome-loaded Secretome/FG might promote surgical anastomosis healing. To verify this, we established a rat model of ischemic ileal anastomosis to test the therapeutic effects. Figure 4A shows a schematic of groups and the experimental design for a 7 days animal study. Figures 4B, C present schematic drawings of blood vessel ligation to induce ischemia in intestinal anastomosis.

Firstly, in order to determine the optimal therapeutic concentration of MSCs secretome loaded into fibrin glue, we conducted animal experiments to screen the therapeutic dose. The secretome of MSCs was loaded into fibrin glue at various concentrations (0-, 1.25-, 2.5-, 5-, 10-, and 20-fold), and six concentration gradient groups were established. After successful animal modeling, Secretome/FG containing different secretome concentrations was smeared onto the surgical anastomosis of rats using a dual-chamber syringe for treatment. The therapeutic effect was preliminary evaluated after 7 days by measuring the anastomotic bursting pressure of rats (Figure 4D). The results showed that, compared with the fibrin glue group (NC, 0-fold concentration), the anastomotic bursting pressure increased in all other treatment groups except for the 1.25-fold concentration group. Among all the treatment groups, the anastomotic bursting pressure was highest in the 10-fold and 20-fold secretome concentration groups, and no significant difference was noted between these two groups. Therefore, we chose the Secretome/FG containing 10-fold secretome as the candidate for further animal experiments (Figure 4E).

After determining the appropriate therapeutic concentration of MSCs secretome in the Secretome/FG delivery system, we developed a rat model of ischemic ileal anastomosis to evaluate the therapeutic effect of MSCs secretome. The rats were assigned into four groups according to different interventions, including the NC group, Secretome-iv group, FG group, and Secretome/FG group. No death was observed before euthanasia of the rats.

By analyzing changes in body weight after surgery, the results revealed an average increase of 6.6 g in the body weight of the Secretome/FG group, which was higher than that of other groups (Figure 4F). Also, the weight of defecation was significantly increased in the Secretome/FG group as compared to other groups (Figure 4G). It was indicated that the intestinal function and food intake of rats were restored following the treatment with Secretome/FG. On the 7th day after surgery, the rats were dissected, and abdominal adhesions were evaluated using the rating scale at specific sites. The results showed that all groups had varying degrees of abdominal adhesions. Whereas, compared with other groups, the adhesions between the surgical sites were reduced in the Secretome/FG group (Figure 4I). In addition, a pressure sensor was used to record the bursting pressure after slow and continuous air perfusion into the isolated small intestine. The results suggested that the Secretome/FG treatment could strengthen the healing strength of the surgical anastomosis because the bursting pressure of the rats in this group was the highest (Figure 4H).

Granulation tissue formation and collagen deposition are the main factors determining sufficient tissue strength and, thus, anastomotic healing (Thompson, Chang, and Jobe, 2006). Hence, we performed HE staining and MTG staining to assess the granulation tissue regeneration and collagen deposition at the anastomotic site. As shown in Figures 4J, K, the thickness of granulation tissues at the anastomosis was increased, and the collagen deposition was more significant in the Secretome/FG group than in other groups. In fact, the collagen content was also positively correlated with the bursting pressure of surgical anastomosis in all rats (Figure 4L). To sum up, the above-mentioned results illustrated that the Secretome/FG treatment could improve the healing strength of intestinal anastomosis in rats and increase the granulation tissue thickness and collagen content, thus improving the healing of the surgical anastomosis; whereas, the single intravenous injection of MSCs secretome exhibited no promoting effect on the anastomotic healing (Figures 4H, J, K).

### 3.4 Secretome/FG creates a beneficial microenvironment for the anastomotic healing

**FIGURE 5:**
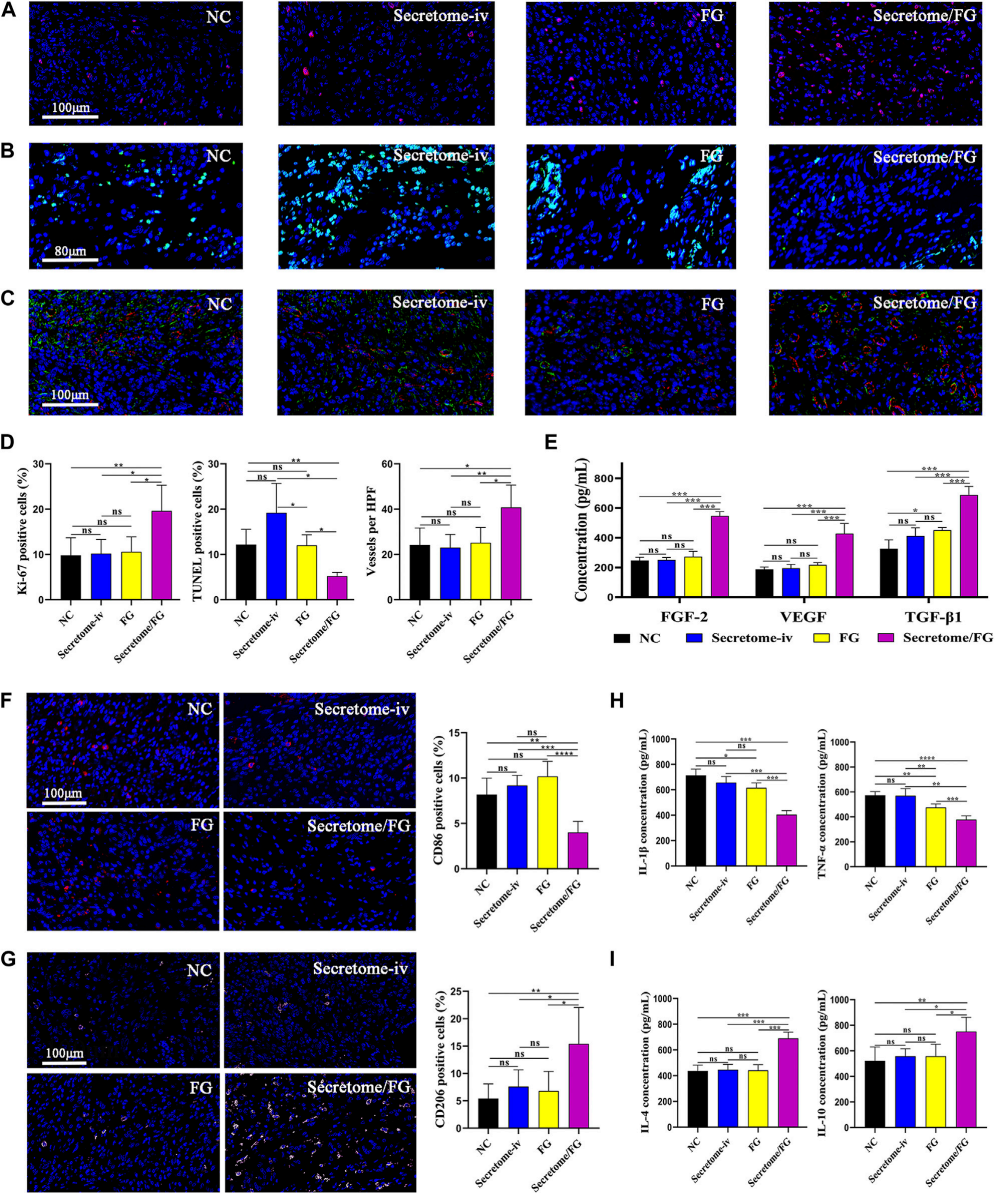
Secretome/FG creates a beneficial tissue microenvironment for the healing of intestinal anastomosis. **(A, B, and C)** Immunofluorescence staining of proliferation, apoptosis, and neovessel formation in regenerated granulation tissues for the different interventions. **(D)** Quantitative analysis of proliferation **(A)**, apoptosis **(B)**, and neovessel formation **(C)** (*n* = 5 per group; ANOVA). **(E)** Quantitative analysis of FGF-2, VEGF, and TGF-β1 in regenerated granulation tissues by ELISA for the different interventions (*n* = 5 per group; ANOVA). **(F, G)** The immunofluorescence staining results show that the Secretome/FG treatment achieves the lowest proportion of M1 macrophage polarization (CD86) and the highest proportion of M2 macrophage polarization (CD206) in the regenerated granulation tissues among all of the interventions. **(H, I)** ELISA quantification of pro-inflammatory factor (IL-1β, TNF-α) and anti-inflammatory factor (IL-4, IL-10) in regenerated granulation tissues for the different interventions (*n* = 5 per group; ANOVA). ****p < 0.0001, ***p < 0.001, **p < 0.01, *p < 0.05, ns ≥ 0.05.

To reveal the microenvironment changes of intestinal anastomotic tissues after the treatment of secretome-loaded Secretome/FG, we conducted immunofluorescence and ELISA to examine the phenotypic changes in surgical anastomotic tissues closely related to tissue repair after different interventions, such as tissue proliferation, angiogenesis, and macrophage polarization. We first analyzed the expression of the proliferation protein Ki67 in anastomotic tissues to evaluate the proliferation surrounding the anastomosis. Immunofluorescence results exhibited that Ki67 was expressed at the highest level in the anastomotic tissues of the Secretome/FG group in comparison with the Secretome-iv, FG, and NC groups (Figure 5A). These results suggested that the Secretome/FG treatment boosted cell proliferation at the anastomotic site. Additionally, we also assessed the cell apoptosis in anastomotic tissues using TUNEL staining and found that the Secretome/FG treatment reduced cell apoptosis in tissues (Figure 5B).

As local hypoxia caused by tissue damage might affect collagen synthesis and degradation, blood perfusion is critical for the repair and protection of anastomotic tissue (Strowitzki et al., 2019). To evaluate angiogenesis, we performed a quantitative analysis of new blood vessels formed in the regenerated granulation tissues through immunofluorescent staining for CD31, a vascular biomarker. The results suggested that a large number of new blood vessels were formed after treatment with Secretome/FG (Figures 5C, D). To further validate the promoting role of Secretome/FG in cell proliferation and angiogenesis, we examined the expression of growth factors VEGF, TGF-β1, and FGF-2 in anastomotic tissues, which are key effector factors to promote cell proliferation, collagen synthesis, and angiogenesis. The ELISA results displayed the highest VEGF, TGF-β1, and FGF-2 levels in the Secretome/FG group (Figure 5E).

Excessive inflammatory cells can degrade collagen in intestinal anastomosis, and macrophages exert a crucial regulatory role in anastomotic inflammation (Thompson, Chang, and Jobe, 2006). Given the distinct cellular functions of classically activated macrophages (pro-inflammatory, M1) and alternatively activated macrophages (wound healing, M2), we examined macrophage polarization infiltrating into the anastomotic tissues through CD86 immunofluorescent staining and CD206 immunofluorescent staining, respectively. The result showed that the Secretome/FG treatment achieved the lowest proportion of M1 macrophage polarization (CD86) and the highest proportion of M2 macrophage polarization (CD206) in the regenerated granulation tissues among all of the interventions (Figures 5F, G). We then measured the expression of inflammatory factors in anastomotic tissues to further validate the anti-inflammatory and pro-reparative effects of Secretome/FG treatment, because pro- and anti-inflammatory cytokines are key effector factors underlying macrophage immunomodulatory effects. ELISA results showed that the Secretome/FG treatment reduced the levels of pro-inflammatory IL-1β and TNF-α and elevated the levels of anti-inflammatory IL-4 and IL-10 in the anastomosis (Figures 5H, I).

Collectively, these results demonstrated that the secretome-loaded Secretome/FG delivery system could facilitate cell proliferation and angiogenesis *in vivo*, regulate macrophage M2 polarization, reduce inflammatory reaction and cell apoptosis surrounding the anastomosis, and increase collagen synthesis, offering a beneficial microenvironment for rapid anastomotic healing.

## 4 Discussion

To our knowledge, this study was the first to investigate the application of MSCs derivatives in intestinal anastomotic repair. This study is mainly aimed at investigating the potential application of MSCs secretome-based therapy in surgical anastomosis. In this study, In order to apply MSCs secretome to the local anastomotic site to exert therapeutic effects, we developed a fibrin glue delivery system loaded with MSCs-derived secretome (Secretome/FG), which could rapidly form a stable intestinal patch *in situ* in the intestinal anastomosis after application. Our results showed that the Secretome/FG delivery system could significantly improve anastomotic healing *via* sustained release of the bioactive factors from the MSCs secretome. Specifically, upon application of the Secretome/FG at surgical anastomosis in rats, the released bioactive factors showed strong promoting effects on anastomotic healing by elevating anastomotic bursting pressure and increasing anastomotic granulation tissue regeneration and collagen deposition. Mechanistically, the MSCs secretome could enhance cell proliferation and angiogenesis, regulate the local immune response, and reduce cell apoptosis, yielding a favorable (promoting wound healing) microenvironment in the intestinal anastomosis.

The utility of stem cell technology for the repair and regeneration of the injured intestine has been a major focus, and stem cell-based regenerative therapies hold promise for anastomotic healing (Flatres et al., 2019; Lightner, 2019; Kent et al., 2021; Zhang et al., 2022). In fact, studies have reported the utilization of stem cell patches in animal experiments to promote anastomotic healing with positive outcomes (Maruya et al., 2017; Van de Putte et al., 2017; Sukho et al., 2018; Morgan et al., 2020; Pan et al., 2020). To date, however, no stem cell-related technology has yet been implemented into clinical practice in the field of anastomotic healing, and its application may be limited by tumorigenic risk as well as low retention and survival post-transplantation (Deng et al., 2018; Nathan and Ustun, 2019). A large body of evidences have indicated that MSCs can play a significant role in tissue repair and regeneration by releasing paracrine factors such as cytokines, chemokines, hormones, and exosomes (Lee et al., 2017; Rahimi et al., 2021; Chouw et al., 2022; Lei et al., 2022; Tang et al., 2022), which are secreted into the culture medium *in vitro* known as “conditioned medium” or “secretome”. As a cell-free technique, transplantation of MSCs secretome is more convenient, safer, cost-effective, and easier to preserve than transplantation of MSCs, with greater potential for clinical translation (Rhatomy et al., 2020). It is also the key reason why we chose the MSCs secretome as a therapeutic agent in our study. We harvested the secretome from the culture medium of MSCs and subsequently detected several growth factors that have been reported to promote surgical anastomotic healing, such as FGF-2, VEGF, and IGF-1 (Oines et al., 2014; Li et al., 2017), which are essential key factors involved in tissue repair and regeneration. *In vitro* studies also found that the MSCs secretome accelerated the viability of key cells involved in tissue repair in a dose-dependent manner. In the rat animal model, the secretome-loaded Secretome/FG delivery system showed strong promoting effects on healing by increasing collagen deposition surrounding the anastomosis. Similar to the promoting role of the MSCs secretome in organ repair after damage such as myocardial infarction (Tang et al., 2022), skin wounds (Lin et al., 2021), and renal injury (Yim et al., 2019), our results also demonstrated the efficacy of the MSCs secretome in improving anastomotic healing.

To improve the utilization efficiency and targetability of drugs, the application of biomaterials as drug delivery vehicles has been extensively studied (Wang et al., 2022). Fibrin glue imitates the final step of the clotting process, and because of its rapid *in situ* gelation properties, it can adhere to the tissue interface, forming a natural barrier. As a result, fibrin glue has been widely used as a hemostatic or tissue sealing agent in clinical practice for decades. In addition, to further ensure safety, tissue adhesion and gelation properties, a commercially available fibrin glue (fibrin adhesive) was used (Spicer and Mikos, 2010; Miller et al., 2019). Recently, fibrin glue has been investigated as a topical delivery carrier for growth factors or drugs, showing positive efficacy by releasing these drug proteins (Willerth et al., 2007; Whelan, Caplice, and Clover, 2014; Viale et al., 2018). Herein, to ensure safety and gelation properties, we used a commercially available fibrin glue as the carrier of MSCs secretome. Our results demonstrated that controlled delivery of the MSCs secretome through fibrin glue seemed to be an effective approach to promote surgical anastomotic repair. Firstly, our *in vitro* experiments showed that MSCs secretome-loaded fibrin glue could sustainably release FGF-2, VEGF, and IGF-1 for up to 10 days, which was similar to the findings of other studies that fibrin glue can exert therapeutic effects through the sustained release of growth factors for extended periods (Tredwell et al., 2006; Yang et al., 2009; Zhou et al., 2011; Al Kayal et al., 2022). The results of subsequent animal experiments suggested its good regenerative potential for repair and also validated fibrin glue as an effective carrier for MSCs secretome. Interestingly, intravenous administration of MSCs secretome alone did not improve anastomotic healing, and at least 10-fold intravenous administration of MSCs secretome was not as effective as topical delivery of fibrin glue loaded with the same MSCs secretome dose. It is well known that drugs administered intravenously have some limitations: 1. Susceptibility to degradation by the complex microenvironment of the circulatory system; 2. Lack of disease targeting; and 3. Poor bioavailability, often requiring an increased drug dose or frequency of administration to be therapeutically effective. This may mainly explain why the intravenous secretome (100 μL in total) did not work *in vivo* in this work. Perhaps increasing the dose or frequency of drug administration would have led to different experimental results. Meanwhile, it also revealed that the local drug delivery system described in our work has more therapeutic advantages than previous studies (Oines et al., 2014) that reported using intravenous drug administration for anastomotic repair. Since the application of the MSCs secretome for anastomosis has not been previously investigated, it has been difficult to make direct comparisons of the efficacy of different modes of administration with other studies. However, other similar studies indirectly confirmed the plausibility of our findings. In a rat model of renal ischemia-reperfusion, Tarng et al. (Tarng et al., 2016) observed improvement of the renal function only in the treatment group that was intravenously injected with highly concentrated 50-fold secretome (4 mL in total). Also, Van Koppen et al. (Van Koppen et al., 2012) observed significant changes in renal function after injection with high-dose 25-fold secretome (2 mL in total) only. However, Hyung et al. reported that, after local injection of the 100-fold or 25-fold secretome (200 μL 10-fold concentrated secretome)-loaded biomaterial into the kidney, a significant improvement was observed in renal function. These results showed the importance of the topical sustained release of bioactive factors and also implied that the topical use of biomaterials at the anastomosis for effective controlled delivery of MSCs secretome seemed to be a requisite for improving anastomotic healing.

In fact, studies in the field of intestinal anastomotic healing have employed fibrin glue as the scaffold to deliver exogenous growth factors VEGF (Li et al., 2017), and growth hormone (Li et al., 2007; Wang et al., 2009), demonstrating fibrin glue’s application potential as a sustained-release carrier for growth factors. However, compared with MSCs secretome therapy, this kind of mono-factor therapy has significant disadvantages. At first, this therapy is not regulated by endogenous negative feedback mechanisms and has the potential to cause tissue hyperproliferation and carcinogenesis (Vajanto et al., 2002; Li et al., 2017; Tamburello et al., 2022). Secondly, this therapy has limited efficacy because tissue repair is accomplished by the concerted action of multiple factors, whereas MSCs secretome therapy has the advantage of containing numerous bioactive factors (Rhatomy et al., 2020). This was also substantiated by our *in vitro* and *in vivo* findings that the MSCs secretome promoted the proliferation of various cells involved in tissue repair, and the numerous bioactive factors released in animal models formed a microenvironment beneficial for granulation tissue formation and collagen synthesis.

As MSCs secretome and fibrin glue have been widely applied as a regenerative drug or tool in various diseases (Spicer and Mikos, 2010; Shin et al., 2018; Miller et al., 2019), our MSCs secretome-loaded fibrin glue delivery system could be easily modified for more interesting biological applications, thus offering great clinical therapeutic potential. In addition to the aforementioned advantage that the MSCs secretome itself is rich in multiple bioactive factors (Rhatomy et al., 2020), the MSCs secretome could be rapidly prepared and stored while being cost-effective as a therapeutic strategy, showing advantages in clinical applications. Furthermore, fibrin glue is a commercialized biomaterial that can be prepared into a variety of agents such as applicators, injections, or sprays depending on clinical treatment needs. Treatment with a combination of MSCs secretome and fibrin glue could contribute to better and faster recovery of patients from surgical anastomosis than before, thus reducing surgical risks and hospital costs.

At last, several limitations existed in this study. Although we identified that the MSCs secretome could promote tissue regeneration, the exact beneficial components of the secretome and the potential mechanisms of intestinal regeneration warrant additional investigations. A comprehensive understanding of the therapeutic efficacy of the MSCs secretome in intestinal regeneration will contribute to a more successful clinical translation of this approach. Next, there is still potential for further improvement in the controlled release properties of the fibrin-based scaffolds used in this study. The development of advanced materials with better MSCs secretome loading techniques and release properties may improve the therapeutic efficacy of secretome. Additionally, the rat model used here could not completely simulate the clinical scenario, and measures such as the mode of intestinal anastomosis and perioperative management all differ from clinical practice, leading to the possibility of some differences in the results. In the future, we intend to conduct experiments in porcine models to explore the application potential of a combined strategy based on the MSCs secretome and biomaterials for anastomotic healing, laying the foundation for future clinical trials.

## 5 Conclusion

MSCs secretome-based therapy is a novel and promising cell-free therapeutic strategy for tissue repair, but its role and reliable delivery system in surgical anastomotic healing remain unknown. Herein, we developed a fibrin glue delivery system loaded with MSCs secretome (Secretome/FG) to induce collagen deposition, cell proliferation, and angiogenesis and regulate macrophage polarization in surgical anastomosis *via* sustained release of bioactive factors in MSCs secretome, which provided a basis for the repair of surgical anastomotic damage. We demonstrated for the first time that the MSCs secretome could promote the healing of intestinal anastomosis. Because the raw material was easily available, simple to use, and biocompatible, this approach can be easily translated into clinical settings. Conclusively, this work demonstrated that this MSCs secretome-based cell-free therapy had significant potential for the rapid repair of intestinal anastomosis.

## Date availability statement

The original contributions presented in the study are included in the article/supplementary material, further inquiries can be directed to the corresponding author.

## References

[B1] Al Kayal T. Buscemi M. Cavallo A. Foffa I. Soldani G. Losi P. (2022). Plasminogen-loaded fibrin scaffold as drug delivery system for wound healing applications. Pharmaceutics 14, 251. 10.3390/pharmaceutics14020251 35213982 PMC8879571

[B2] Chen W. Sun Y. Gu X. Cai J. Chen S. Zhang X. (2021). Conditioned medium of human bone marrow-derived stem cells promotes tendon-bone healing of the rotator cuff in a rat model. Biomaterials 271, 120714. 10.1016/j.biomaterials.2021.120714 33610048

[B3] Chouw A. Milanda T. Sartika C. R. Kirana M. N. Halim D. Faried A. (2022). Potency of mesenchymal stem cell and its secretome in treating COVID-19. Regen. Eng. Transl. Med. 8, 43–54. 10.1007/s40883-021-00202-5 33723519 PMC7945610

[B4] Deng J. Zhang Y. Xie Y. Zhang L. Tang P. (2018). Cell transplantation for spinal cord injury: Tumorigenicity of induced pluripotent stem cell-derived neural stem/progenitor cells, Stem Cells Int., 2018, 1–7. 10.1155/2018/5653787 PMC5817265 29535771

[B5] Flatres C. Loffet E. Neunlist M. Mahe M. M. (2019). Façonner l’intestin à partir des cellules souches pluripotentes humaines. Med. Sci. Paris. 35, 549–555. 10.1051/medsci/2019096 31274085

[B6] Hansmann G. Chouvarine P. Diekmann F. Giera M. Ralser M. Mülleder M. (2022). Human umbilical cord mesenchymal stem cell-derived treatment of severe pulmonary arterial hypertension. Nat. Cardiovasc Res. 1, 568–576. 10.1038/s44161-022-00083-z PMC11358026 39195868

[B7] Huang J. Ren H. Jiang Y. Wu X. Ren J. (2020). Technique advances in enteroatmospheric fistula isolation after open abdomen: A review and outlook. Front. Surg. 7, 559443. 10.3389/fsurg.2020.559443 33553237 PMC7855170

[B8] Kent I. Freund M. Agarwal S. Wexner S. (2021). The application of regenerative medicine in colorectal surgery. Surgery 171, 867–872. 10.1016/j.surg.2021.08.026 34649714

[B9] Khan O. Nizar S. Vasilikostas G. Wan A. (2012). Minimally invasive versus open oesophagectomy for patients with oesophageal cancer: A multicentre, open-label, randomised controlled trial. J. Thorac. Dis. 4, 465–466. 10.3978/j.issn.2072-1439.2012.08.16 23050109 PMC3461074

[B10] Kiran R. P. Murray A. Chiuzan C. Estrada D. Forde K. (2015). Combined preoperative mechanical bowel preparation with oral antibiotics significantly reduces surgical site infection, anastomotic leak, and ileus after colorectal surgery. Ann. Surg. 262, 416–425. 10.1097/SLA.0000000000001416 26258310

[B11] Lee S. Y. Kwon B. Lee K. Son Y. H. Chung S. G. (2017). Therapeutic mechanisms of human adipose-derived mesenchymal stem cells in a rat tendon injury model. Am. J. Sports Med. 45, 1429–1439. 10.1177/0363546517689874 28291954

[B12] Lei F. Li M. Lin T. Zhou H. Wang F. Su X. (2022). Treatment of inflammatory bone loss in periodontitis by stem cell-derived exosomes. Acta biomater. 141, 333–343. 10.1016/j.actbio.2021.12.035 34979326

[B13] Li Y. Bao Y. Jiang T. Tan L. Liu F. Li J. (2007). Combination of fibrin glue with growth hormone augments healing of incomplete intestinal anastomoses in a rat model of intra-abdominal sepsis: A dynamic study. J. Invest. Surg. 20, 301–306. 10.1080/08941930701598826 17972218

[B14] Li Z. Wang W. Wang X. Jiang L. Wang F. Liu Q. (2017). Sustained-released mixture of vascular endothelial growth factor 165 and fibrin glue strengthens healing of ileal anastomoses in a rabbit model with intraperitoneal infection. Ann. Surg. Treat. Res. 93, 159–165. 10.4174/astr.2017.93.3.159 28932732 PMC5597540

[B15] Liang X. Li J. Yan Y. Xu Y. Wang X. Wu H. (2022). Efficacy of microneedling combined with local application of human umbilical cord-derived mesenchymal stem cells conditioned media in skin brightness and rejuvenation: A randomized controlled split-face study. Front. Med. (Lausanne) 9, 837332. 10.3389/fmed.2022.837332 35685406 PMC9171013

[B16] Lightner A. L. (2019). Stem cell therapies for inflammatory bowel disease. Curr. Gastroenterol. Rep. 21, 16. 10.1007/s11894-019-0672-y 30955111

[B17] Lin H. Chen H. Zhao X. Chen Z. Zhang P. Tian Y. (2021). Advances in mesenchymal stem cell conditioned medium-mediated periodontal tissue regeneration. J. Transl. Med. 19, 456. 10.1186/s12967-021-03125-5 34736500 PMC8567704

[B18] Maruya Y. Kanai N. Kobayashi S. Koshino K. Okano T. Eguchi S. (2017). Autologous adipose-derived stem cell sheets enhance the strength of intestinal anastomosis. Regen. Ther. 7, 24–33. 10.1016/j.reth.2017.06.004 30271849 PMC6134898

[B19] Miller R. Wormald J. C. R. Wade R. G. Collins D. P. (2019). Systematic review of fibrin glue in burn wound reconstruction. Br. J. Surg. 106, 165–173. 10.1002/bjs.11045 30724361

[B20] Morgan A. Zheng A. Linden K. M. Zhang P. Brown S. A. Carpenter J. P. (2020). Locally transplanted adipose stem cells reduce anastomotic leaks in ischemic colorectal anastomoses: A rat model. Dis. Colon Rectum 63, 955–964. 10.1097/DCR.0000000000001667 32168095

[B21] Nathan S. Ustun C. (2019). Complications of stem cell transplantation that affect infections in stem cell transplant recipients, with analogies to patients with hematologic malignancies. Infect. Dis. Clin. North Am. 33, 331–359. 10.1016/j.idc.2019.01.002 30940464

[B22] Oines M. N. Krarup P. M. Jorgensen L. N. Agren M. S. (2014). Pharmacological interventions for improved colonic anastomotic healing: A meta-analysis. World J. Gastroenterol. 20, 12637–12648. 10.3748/wjg.v20.i35.12637 25253969 PMC4168102

[B23] Pan H. Lam P. K. Tong S. W. Leung K. K. Teoh A. Y. Ng E. K. (2020). Mesenchymal stem cells combined with tissue fusion technology promoted wound healing in porcine bowel anastomosis. Stem Cells Int. 2020, 1–14. 10.1155/2020/5142797 PMC7038387 32104185

[B24] Rahimi B. Panahi M. Saraygord-Afshari N. Taheri N. Bilici M. Jafari D. (2021). The secretome of mesenchymal stem cells and oxidative stress: Challenges and opportunities in cell-free regenerative medicine. Mol. Biol. Rep. 48, 5607–5619. 10.1007/s11033-021-06360-7 34191238

[B25] Reischl S. Wilhelm D. Friess H. Neumann P. A. (2021). Innovative approaches for induction of gastrointestinal anastomotic healing: An update on experimental and clinical aspects. Langenbecks Arch. Surg. 406, 971–980. 10.1007/s00423-020-01957-1 32803330 PMC8208906

[B26] Rhatomy S. Prasetyo T. E. Setyawan R. Soekarno N. R. Romaniyanto F. Sedjati A. P. (2020). Prospect of stem cells conditioned medium (secretome) in ligament and tendon healing: A systematic review. Stem Cells Transl. Med. 9, 895–902. 10.1002/sctm.19-0388 32304180 PMC7381802

[B27] Rose J. Weiser T. G. Hider P. Wilson L. Bickler S. W. (2015). Estimated need for surgery worldwide based on prevalence of diseases: A modelling strategy for the WHO global Health estimate. Lancet Glob. Health 3, S13–S20. 10.1016/S2214-109X(15)70087-2 25926315 PMC5746187

[B28] Shin H. Chong H. W. Chung W. K. Park B. S. (2018). Up-to-date clinical trials of hair regeneration using conditioned media of adipose-derived stem cells in male and female pattern hair loss. Curr. Stem Cell Res. Ther. 12, 524–530. 10.2174/1574888X12666170504120244 28474542

[B29] Spicer P. P. Mikos A. G. (2010). Fibrin glue as a drug delivery system. J. Control Release 148, 49–55. 10.1016/j.jconrel.2010.06.025 20637815 PMC3005546

[B30] Strowitzki M. J. Ritter A. S. Kimmer G. Schneider M. (2019). Hypoxia-adaptive pathways: A pharmacological target in fibrotic disease? Pharmacol. Res. 147, 104364. 10.1016/j.phrs.2019.104364 31376431

[B31] Sukho P. Boersema G. S. A. Kops N. Lange J. F. Kirpensteijn J. Hesselink J. W. (2018). Transplantation of adipose tissue-derived stem cell sheet to reduce leakage after partial colectomy in A rat model. J. Vis. Exp. 138, 57213. 10.3791/57213 PMC6126783 30148499

[B32] Sushmitha S. Antara B. Rosa Di Jothimani G. Gopinath M. Murugesan R. (2018). Concise review on clinical applications of conditioned medium derived from human umbilical cord-mesenchymal stem cells (UC-MSCs). Int. J. Hematol. Oncol. Stem Cell Res. 12, 230–234. 30595826 PMC6305261

[B33] Tamburello M. Altieri B. Sbiera I. Sigala S. Berruti A. Fassnacht M. (2022). FGF/FGFR signaling in adrenocortical development and tumorigenesis: Novel potential therapeutic targets in adrenocortical carcinoma. Endocrine 77, 411–418. 10.1007/s12020-022-03074-z 35583844 PMC9385797

[B34] Tang J. Cui X. Zhang Z. Xu Y. Guo J. Soliman B. G. (2022). Injection-free delivery of MSC-derived extracellular vesicles for myocardial infarction therapeutics. Adv. Healthc. Mater 11, e2100312. 10.1002/adhm.202100312 34310068

[B35] Tarng D. C. Tseng W. C. Lee P. Y. Chiou S. H. Hsieh S. L. (2016). Induced pluripotent stem cell-derived conditioned medium attenuates acute kidney injury by downregulating the oxidative stress-related pathway in ischemia-reperfusion rats. Cell Transpl. 25, 517–530. 10.3727/096368915X688542 26132529

[B36] Thompson S. K. Chang E. Y. Jobe B. A. (2006). Clinical review: Healing in gastrointestinal anastomoses, part I. Microsurgery 26, 131–136. 10.1002/micr.20197 16518804

[B37] Tredwell S. Jackson J. K. Hamilton D. Lee V. Burt H. M. (2006). Use of fibrin sealants for the localized, controlled release of cefazolin. Can. J. Surg. 49, 347–352. 17152573 PMC3207579

[B38] Vajanto I. Rissanen T. T. Rutanen J. Hiltunen M. O. Tuomisto T. T. Arve K. (2002). Evaluation of angiogenesis and side effects in ischemic rabbit hindlimbs after intramuscular injection of adenoviral vectors encoding VEGF and LacZ. J. Gene Med. 4, 371–380. 10.1002/jgm.287 12124979

[B39] Van de Putte D. Demarquay C. Van Daele E. Moussa L. Vanhove C. Benderitter M. (2017). Adipose-derived mesenchymal stromal cells improve the healing of colonic anastomoses following high dose of irradiation through anti-inflammatory and angiogenic processes. Cell Transpl. 26, 1919–1930. 10.1177/0963689717721515 PMC5802630 29390877

[B40] van Koppen A. Joles J. A. van Balkom B. W. Lim S. K. de Kleijn D. Giles R. H. (2012). Human embryonic mesenchymal stem cell-derived conditioned medium rescues kidney function in rats with established chronic kidney disease. Plos One 7, e38746. 10.1371/journal.pone.0038746 22723882 PMC3378606

[B41] Viale M. Monticone M. Maric I. Giglio V. Profumo A. Aprile A. (2018). Characterization of drug release from fibrin gels loaded with different pharmaceutical and experimental doxorubicin formulations. Pharmacol. Rep. 70, 760–765. 10.1016/j.pharep.2018.02.014 29936363

[B42] Wang C. Min J. B. Kim S. N. Park W. Park H. H. Kim T. H. (2022). Biomaterials as therapeutic drug carriers for inflammatory bowel disease treatment. J. Control Release 345, 1–19. 10.1016/j.jconrel.2022.02.028 35227764

[B43] Wang F. Ren J. Wang G. Ren H. Hong Z. Wu X. (2019). Early active drainage by fine tube bundles improves the clinical outcome of anastomotic leak after abdominal surgery: A pilot randomized, controlled trial in two tertiary hospitals in China. Surg. Infect. (Larchmt) 20, 208–214. 10.1089/sur.2018.177 30614767

[B44] Wang P. Wang J. Zhang W. Li Y. Li J. (2009). Effect of the combination of fibrin glue and growth hormone on intestinal anastomoses in a pig model of traumatic shock associated with peritonitis. World J. Surg. 33, 567–576. 10.1007/s00268-008-9889-x 19132439

[B45] Whelan D. Caplice N. M. Clover A. J. (2014). Fibrin as a delivery system in wound healing tissue engineering applications. J. Control Release 196, 1–8. 10.1016/j.jconrel.2014.09.023 25284479

[B46] Willerth S. M. Johnson P. J. Maxwell D. J. Parsons S. R. Doukas M. E. Sakiyama-Elbert S. E. (2007). Rationally designed peptides for controlled release of nerve growth factor from fibrin matrices. J. Biomed. Mater Res. A 80, 13–23. 10.1002/jbm.a.30844 16958043

[B47] Yang P. Wang C. Shi Z. Huang X. Dang X. Xu S. (2009). Prefabrication of vascularized porous three-dimensional scaffold induced from rhVEGF<sub>165</sub>: A preliminary study in rats. Cells Tissues Organs 189, 327–337. 10.1159/000142162 18587233

[B48] Yim H. E. Kim D. S. Chung H. C. Shing B. Moon K. H. George S. K. (2019). Controlled delivery of stem cell-derived trophic factors accelerates kidney repair after renal ischemia-reperfusion injury in rats. Stem Cells Transl. Med. 8, 959–970. 10.1002/sctm.18-0222 31144785 PMC6708069

[B49] Zhang H. M. Yuan S. Meng H. Hou X. T. Li J. Xue J. C. (2022). Stem cell-based therapies for inflammatory bowel disease. Int. J. Mol. Sci. 23, 8494. 10.3390/ijms23158494 35955628 PMC9368934

[B50] Zhou W. Zhao M. Zhao Y. Mou Y. (2011). A fibrin gel loaded with chitosan nanoparticles for local delivery of rhEGF: Preparation and *in vitro* release studies. J. Mater Sci. Mater Med. 22, 1221–1230. 10.1007/s10856-011-4304-9 21445654

